# Apocarotenoids Involved in Plant Development and Stress Response

**DOI:** 10.3389/fpls.2019.01168

**Published:** 2019-09-27

**Authors:** Abrar Felemban, Justine Braguy, Matias D. Zurbriggen, Salim Al-Babili

**Affiliations:** ^1^The BioActives Lab, Biological and Environmental Sciences and Engineering Division, King Abdullah University of Science and Technology (KAUST), Thuwal, Saudi Arabia; ^2^Institute of Synthetic Biology and CEPLAS, University of Düsseldorf, Düsseldorf, Germany

**Keywords:** apocarotenoids, abscisic acid (ABA), strigolactones (SLs), β-cyclocitral, zaxinone, anchorene

## Abstract

Carotenoids are isoprenoid pigments synthesized by all photosynthetic organisms and many heterotrophic microorganisms. They are equipped with a conjugated double-bond system that builds the basis for their role in harvesting light energy and in protecting the cell from photo-oxidation. In addition, the carotenoids polyene makes them susceptible to oxidative cleavage, yielding carbonyl products called apocarotenoids. This oxidation can be catalyzed by carotenoid cleavage dioxygenases or triggered nonenzymatically by reactive oxygen species. The group of plant apocarotenoids includes important phytohormones, such as abscisic acid and strigolactones, and signaling molecules, such as β-cyclocitral. Abscisic acid is a key regulator of plant’s response to abiotic stress and is involved in different developmental processes, such as seed dormancy. Strigolactone is a main regulator of plant architecture and an important signaling molecule in the plant-rhizosphere communication. β-Cyclocitral, a volatile derived from β-carotene oxidation, mediates the response of cells to singlet oxygen stress. Besides these well-known examples, recent research unraveled novel apocarotenoid growth regulators and suggests the presence of yet unidentified ones. In this review, we describe the biosynthesis and biological functions of established regulatory apocarotenoids and touch on the recently identified anchorene and zaxinone, with emphasis on their role in plant growth, development, and stress response.

## Introduction

Carotenoids are a large group of isoprenoid pigments, which includes more than 600 distinct compounds (Britton et al., 2012). Carotenoids are present in all clades of life; however, their synthesis is restricted to photosynthetic organisms and some nonphotosynthetic fungi and bacteria (Ruiz-Sola and Rodríguez-Concepción, 2012; Moise et al., 2014; Nisar et al., 2015). In plants, carotenoids are essential components of the photosynthetic apparatus where they act as photoprotective pigments and contribute to the light harvesting process. In addition, plant carotenoids confer their bright orange, reddish, or yellow colors to many fruits and flowers (DellaPenna and Pogson, 2006; Ahrazem et al., 2016a). Animals are not able to synthesize carotenoids de novo and rely on nutritional sources to cover their needs of these pigments that act as vitamin A precursor and antioxidants (Fraser and Bramley, 2004).

The electron-rich polyene system of carotenoids makes them susceptible to oxidation processes that break the carotenoid backbone. This cleavage reaction is catalyzed by carotenoid cleavage dioxygenases (CCDs), which build a ubiquitous family of non-heme iron enzymes, and leads to products called apocarotenoids (Giuliano et al., 2003; Hou et al., 2016). Apocarotenoids can also arise through non-enzymatic attack by reactive oxygen species (ROS). In both cases, apocarotenoids fulfill, with or without further enzymatic modifications, many important biological functions. For instance, these metabolites act as precursors of the phytohormones abscisic acid (ABA) and strigolactones (SLs), which are best known for their role in abiotic stress response and adapting plant architecture to nutrition availability, respectively (Jia et al., 2017). Other apocarotenoids are known as signaling molecules regulating cell response to oxidative stress or have been recently discovered as plant growth regulators. In this review, we describe the role of apocarotenoids in plant growth and development and address their biosynthesis and transport, focusing on ABA, SLs, and recently identified regulatory apocarotenoids.

### General Aspects of Apocarotenoid Formation

The formation of apocarotenoids is initiated by oxidative cleavage reactions that can be catalyzed by enzymes, generally CCDs, or take place through exposure of carotenoids to ROS. β-Cyclocitral is an example for a carotenoid cleavage product that can be formed following both scenarios (Ramel et al., 2012b; Rodrigo et al., 2013). The formation of this β-carotene–derived volatile in photosynthetic tissues is triggered by a singlet oxygen ^1^O_2_ attack, which takes place in the photosystem II, particularly under high light stress (D’Alessandro et al., 2019), while in citrus fruits, it is catalyzed by a CCD (CCD4b) that cleaves β-carotene, β-cryptoxanthin, and zeaxanthin (Rodrigo et al., 2013). Although some CCDs show a quite relaxed substrate and site (double bond) specificity (Ilg et al., 2014), cleavage reactions catalyzed by these enzymes generally take place at certain double bond(s) in defined carotenoid/apocarotenoid substrate(s) (Walter and Strack, 2011; McQuinn et al., 2015). It should be also mentioned here that some bacterial and fungal members of the CCD family cleave biphenylic, stilbene substrates instead of carotenoids (Brefort et al., 2011).

The genome of *Arabidopsis* encodes nine members of the CCD family, which includes five 9-*cis*-epoxy carotenoid dioxygenases (NCED2, NCED3, NCED5, NCED6, and NCED9) and four, CCDs (CCD1, CCD4, CCD7, and CCD8). NCED enzymes catalyze the first steps in ABA biosynthesis by cleaving the (C11–C12) double bond in 9-*cis*-violaxanthin and 9′-*cis*-neoxanthin, which yields the ABA precursor xanthoxin (Tan et al., 2003). CCD1 enzymes have the ability to cleave several carotenoid and apocarotenoid substrates—linear, monocycle, and bicycle—at different positions along the carbon backbone (Schwartz et al., 2001; Vogel et al., 2008; Ilg et al., 2009; Ilg et al., 2014), which leads to volatile compounds responsible for aroma and flavor in different plant species. CCD4 is also involved in the formation of volatile compounds by cleaving carotenoids either at the (C7–C8) double bond in cryptoxanthin and zeaxanthin, as shown for *Citrus* CCD4b, or at the (C9–C10) double bond in bicyclic carotenoids, as shown for the *Arabidopsis* and potato CCD4 (Rubio-Moraga et al., 2014; Bruno et al., 2015; Bruno et al., 2016). CCD4 activity regulates carotenoid content in different plant tissues and produces the citrus pigment citraurin. Finally, the sequential action of CCD7 and CCD8 enzymes leads to the SL precursor carlactone. CCD7 cleaves 9-*cis*-β-carotene formed by the carotene isomerase DWARF27 from the corresponding all-*trans*-isomer at the (C9′–C10′) double bound to produce 9-*cis*-β-apo-10′-carotenal and β-ionone (Alder et al., 2012; Bruno et al., 2014; Bruno and Al-Babili, 2016; Haider et al., 2018). CCD8 action follows by converting 9-*cis*-β-apo-10′-carotenal into carlactone the substrate of CYP450 enzymes (711A1 clade) that produce different SLs (Abe et al., 2014; Zhang et al., 2014). A further CCD type (CCD2) has been identified in *Crocus* species, which cleaves the carotenoid chain at the (C7–C8) and (C7′–C8′) double bounds, converting zeaxanthin into crocetin dialdehyde, the precursor of crocin, a saffron pigment, and 3-hydroxy-cyclocitral (Frusciante et al., 2014; Ahrazem et al., 2016b). Very recently, a survey of 748 sequences of all CCDs genes from 69 plant species unraveled an overlooked plant CCD clade represented by the rice zaxinone synthase (ZAS) that cleaves the apocarotenoid apo-10′-zeaxanthinal at the (C13–C14) double bond to produce zaxinone, a novel growth regulator required for normal rice growth (Wang et al., 2019). Recently, the discovery of a new biosynthetic pathway for apocarotenoids was shown to be CCDs independent, through the action of TomLocX/LOX lipoxygenase (in tomato and *Arabidopsis*, respectively) (Gao et al., 2019).

## Abscisic Acid

Abscisic acid is an essential carotenoid-derived plant hormone that coordinates plant response to stress stimuli and is involved in different developmental processes. In addition, ABA regulates water uptake (Dodd, 2013; Harris, 2015) and coordinates stomata closure to minimize water loss under drought condition (Merilo et al., 2015). Furthermore, this hormone controls seed maturation and is responsible for seed dormancy that prevents newly produced seeds from germination under unfavorable conditions (Tuan et al., 2018).

### ABA Metabolism

**Figure 1:**
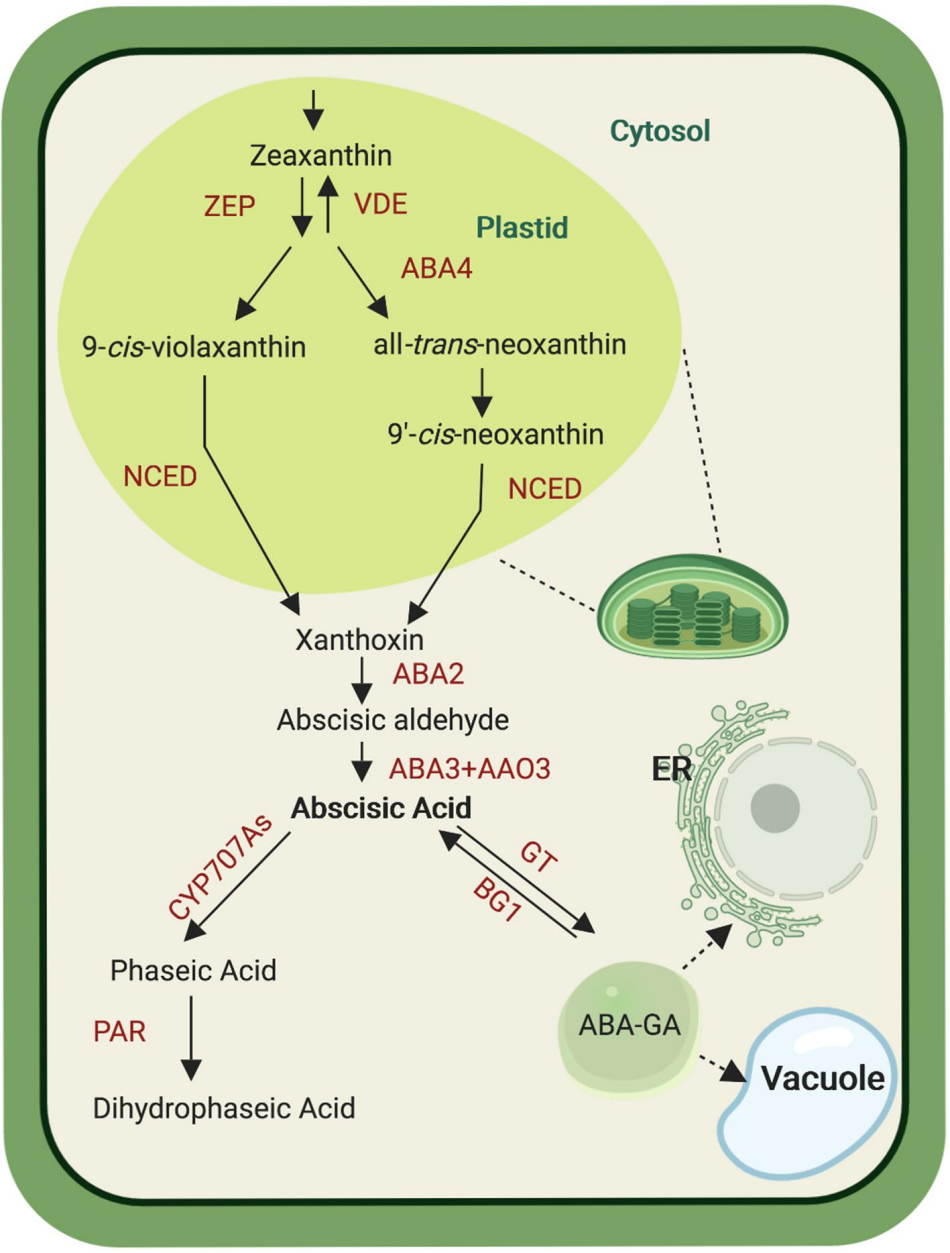
Abscisic acid (ABA) biosynthesis pathway. *De novo* ABA biosynthesis begins in plastid, catalyzed by several enzymes (ZEP, zeaxanthin epoxidase; VDE, violaxanthin de-epoxidase; ABA4, ABA-deficient 4; NCED, nine-*cis*-apoxicarotenoid-dioxygenase ) that convert zeaxanthin into xanthoxin. After transport into the cytosol, xanthoxin is subjected to oxidation steps mediated by three enzymes (ABA2, ABA-deficient 2; AAO3, abscisic aldehyde oxidase and ABA3, ABA-deficient 3) giving rise to ABA. Abscisic acid can be modified by CYP707A enzymes that yield phaseic acid (PA). The latter is the substrate of the PA reductase (PAR) that forms dihydrophaseic acid. Alternatively, ABA is glucosylated by ABA glucosyltransferase (GT) into ABA-GT that can be transported into the vacuole and the endoplasmic reticulum (ER). Abscisic acid–GT can be converted back into ABA by the glycosyl hydrolase BG1. Created with ‘Biorender’.

Plant ABA biosynthesis takes place in different cell compartments, as it starts in plastids and is finalized in the cytosol (**Figure 1**). This metabolic process is initiated by cleaving C_40_ 9-*cis*-epoxycarotenoids, i.e. 9-*cis*-violaxanthin and 9′-*cis*-neoxanthin, which represents the first committed and rate-limiting step in ABA biosynthesis (Finkelstein, 2013). Both xanthophylls, violaxanthin and neoxanthin, are formed from all-*trans*-zeaxanthin by two epoxidation steps catalyzed by zeaxanthin epoxidase, yielding all-*trans*-violaxanthin. These epoxidation reactions can be reversed by violaxanthin de-epoxidase. All-*trans*-violaxanthin is then converted into all-*trans*-neoxanthin in a less understood process that involves the protein ABA-deficient 4 (ABA4) in *Arabidopsis* (North et al., 2007). Both all-*trans*-xanthophylls are isomerized into the required *cis*-forms by yet unclear mechanism(s) (Dejonghe et al., 2018). The following cleavage reaction, catalyzed by NCED, takes place in plastids and yields the C_15_ compound xanthoxin and a C_25_-apocarotenoid. The former is then relocalized from plastids to the cytosol where it becomes available for the final two steps of the ABA biosynthesis. The latter consists of the C_25_-apocarotenoid conversion into abscisic aldehyde by the short-chain dehydrogenase/reductase-like enzyme ABA-deficient 2 (ABA2), followed by the oxidation to ABA by abscisic aldehyde oxidase (AAO) that belongs to a multienzyme family represented by four members in *Arabidopsis* (AAO1, AAO2, AAO3, and AAO4). Mutant analysis points to AAO3 as the major contributor to ABA formation, among these four enzymes (Seo et al., 2000). The AAO activity requires molybdenum cofactor that is formed by the sulfurase ABA3 (Iuchi et al., 2001; Hauser et al., 2017), which is supported by the ABA deficiency in *aba3* loss of function mutants (Watanabe et al., 2018). Abscisic acid can be further metabolized by ABA-8′-hydroxylases, CYP enzymes of the 707A clade, which introduce a hydroxyl-group at the C-8′ position, yielding the instable 8′-OH-ABA that isomerizes spontaneously by internal cyclization to phaseic acid (PA). A soluble reductase converts PA to dihydrophaseic acid (DPA) by reducing the 4′-ketone group. In addition to the C-8′ position, ABA can be hydroxylated at the C-7′ and C-9′ positions. PA and DPA are considered as major ABA catabolites (Hauser et al., 2017; Ma et al., 2018). However, it was recently shown that PA acts also as a hormone in seed plants and can be recognized by some ABA receptors (Weng et al., 2016). Abscisic acid can also be further catabolized through the action of the glucosyltransferase subfamily ABA uridine diphosphate glucosyltransferases into ABA glucosyl esters (ABA-GE). ABA-GE accumulates in vacuoles and is considered as a storage or transport form of ABA, which can be cleaved back into ABA by β-glucosidases under dehydration stress (Lee et al., 2006; Xu et al., 2012; Ma et al., 2018).

### ABA Signaling Pathways

Modulation of plant growth upon exposure to abiotic stress conditions (high salinity, drought, and low temperature) is coordinated by several hormones and proteins, including regulatory factors (Todaka et al., 2015). In *Arabidopsis*, the phytohormone ABA has been shown to be a key player in this process, by activating the ABA-dependent signaling pathway. This pathway consists of three major components: PYR (pyrabactin resistance)/PYL (PYR1-LIKE)/RCAR (regulatory component of ABA response) group of ABA receptors, PP2C (protein phosphatase 2C; negative regulator), and SnRK2 (sucrose nonfermenting 1–related protein kinase 2; positive regulator) (Todaka et al., 2015). Together, these components form an ABA sensing and signal transducing complex: in the absence of ABA, the negative regulator PP2C represses SnRK2 activity by dephosphorylating its kinase activating loop, while in the presence of ABA, the receptors PYR/PYL/RCAR5 and PP2C form a complex that prevents the PP2C-mediated dephosphorylation of SnRK2. The resulting activated SnRK2 phosphorylates and activates ABA-responsive element (ABRE)–binding protein/ABRE-binding factor (AREB/ABF) transcription factors, which bind to promoters of drought responsive genes and trigger plant stress response (Todaka et al., 2015; Wang et al., 2017; Xie et al., 2019). There is also an ABA-independent signaling pathway that regulates plant response to abiotic stress. Dehydration responsive element-binding (DREB) proteins are transcription factors induced by stress conditions, such as high salinity, drought, and heat stress (Todaka et al., 2015). Under high temperature stress, heat shock factor A1 and ABRE-binding proteins/ABRE binding factor 3 (AREB1/AREB2/ABF3) regulate DREB2A expression through the binding to ABRE and heat shock element motifs, in their promoter region, to enhance plant resistance to high temperature (Todaka et al., 2015; Xie et al., 2019).

### ABA Regulates Seed Dormancy and Germination

**Figure 2:**
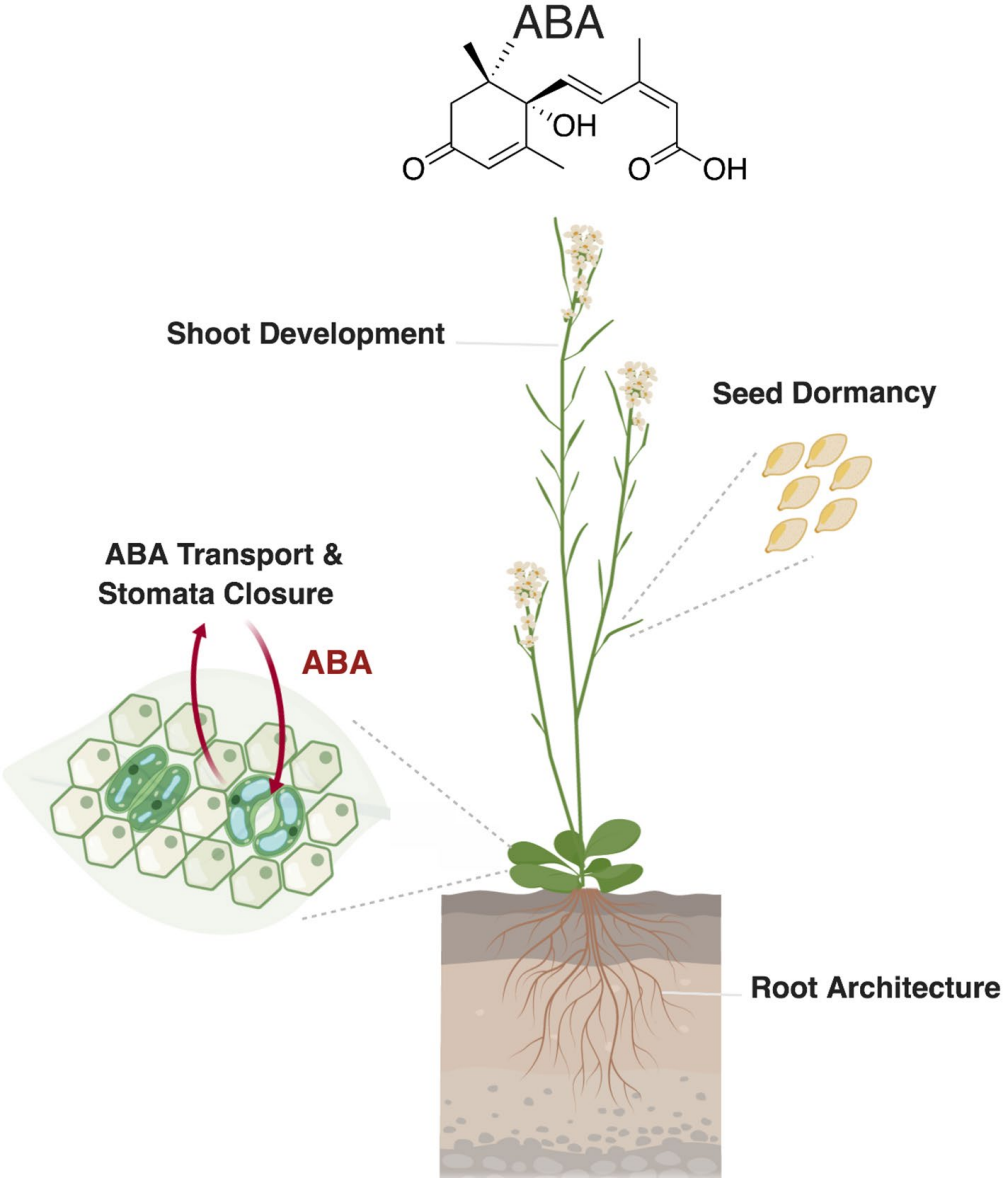
Role of abscisic acid (ABA) in plant development and stress response. Abscisic acid regulates different developmental processes, such as seed dormancy and shoot and root development. In leaves, ABA transport to the guard cells triggers stomatal closure in response to biotic and abiotic stresses. Created with ‘Biorender’.

Seed dormancy is a desirable trait allowing the uniform postharvest germination of seeds under favorable environmental conditions. In *Arabidopsis* seeds, ABA shows two peaks of accumulation, which arise during middle (25 days after pollination [DAP]) and late phases (35 DAP) of seed maturation (Kanno et al., 2010). In rice, it was shown that the accumulation of ABA, which occurs at early and middle stages of seed development (10–20 DAP), induces seed dormancy (Suzuki et al., 2000) and that the decrease of ABA level in seeds results in loss of dormancy. These changes in ABA concentration are determined by the expression of the ABA biosynthesis (NCED) and catabolic genes (CYP707A1and CYP707A2) (**Figure 2**) (Du et al., 2015). The role of CYP707A in reducing ABA content and paving the way for seed germination was also observed in barley. It was shown that the increase in the expression level of HvABA8′OH, a barley CYP707A enzyme, correlated with a reduction of ABA content in ripe seeds. *HvABA8*′*OH1* RNAi lines confirmed the role of this enzyme in ABA catabolism, as they showed enhanced seed dormancy (Gubler et al., 2008). But how does ABA regulate seed dormancy?

Gibberellic acid (GA) is an additional hormone that plays a key role in seed germination. Thus, this developmental process is regulated by a balance between ABA and GA, which act here as antagonists in signaling and biosynthesis pathways (Finkelstein et al., 2008; Née et al., 2017b). In *Arabidopsis*, DELLA proteins act as regulators of ABA and GA response, balancing between dormancy and germination by interacting with different genes. In the absence of GA, DELLAs interact with (i) XERICO, a RING-H2 zinc finger ubiquitin ligase, which enhances ABA accumulation, and (ii) ABA insensitive 5 (ABI5), an ABA-regulated transcription factor, leading to inhibition of seed germination/maintenance of seed dormancy (Zeng et al., 2014; Zeng et al., 2015). Previous studies identified another genetic element, called delay of germination 1 (DOG1), which regulates seed dormancy and germination in *Arabidopsis*, as it interferes with both ABA and GA. Two PP2C phosphatases, ABA-hypersensitive germination 1 (AHG1) and AHG3, have been identified interacting with DOG1 in imbibed and dry seeds and revealed to be essential for DOG1 function in controlling seed dormancy (Née et al., 2017a).

### ABA Regulates Shoot Development

Several lines of evidence suggest a role of ABA in inhibiting shoot branching by maintaining axillary buds dormancy (reviewed in Barbier et al., 2019). Branched 1 (BRC1), a transcription factor of the Teosinte branched 1, Cycloidea, PCF1 (TCP) family, plays an important role in regulating bud outgrowth in response to plant hormones, nutrients availability, and light quality. BRC1 binds and activates three homeodomain leucine zipper (HD-ZIP) transcription factors, which stimulate NCED3 expression. The increase in NCED3 activity results in enhanced ABA level, indicating that ABA enhances bud dormancy downstream of BRC1 (Barbier et al., 2019). In addition, recent results show that ABA is involved in the regulation of shoot branching by light quality and intensity, as ABA accumulation and synthesis in buds negatively correlate with the red to far red-light ratio (R:FR) (Reddy et al., 2013; González-Grandío et al., 2017; Holalu and Finlayson, 2017). Abscisic acid also affects leaf and shoot growth through repressing ethylene production, which maintains leaf expansion and enhances shoot growth (Hussain et al., 2000). A further evidence for the role of ABA in shoot growth is provided by the ABA-deficient mutant line *aba2-1* that shows shoot growth inhibition, a phenotype that can be rescued by exogenous application of this hormone (LeNoble et al., 2004) (**Figure 2**).

### Antagonistic or Synergistic Actions of ABA With Other Phytohormones on the Root System Development

Plants modulate their architecture in response to environmental stimuli. These adaptation processes include also alteration in the root system that shows high sensitivity to environmental changes, particularly to harsh growth conditions. In response to salt stress, ABA stimulates the elongation of primary and lateral roots (Harris, 2015). During drought stress, ABA promotes primary root elongation, allowing the roots to grow deeper to reach water (Hauser et al., 2017). Generally, in well-watered conditions, ABA is known as an inhibitor of root development (Sharp and LeNoble, 2002). However, exogenous ABA application shows that at low concentration (0.1 μM), ABA stimulates root growth, while it exerts the opposite effect when applied at high concentration (10 μM). Thus, ABA acts in a concentration-dependent manner, antagonistically or synergistically with other phytohormones, such as ethylene and auxin, to fine tune plant root architecture. Ethylene represses root growth under stress conditions by inhibiting root cell elongation (Le et al., 2001; Ruzicka et al., 2007; Li et al., 2017). It has been shown that the growth inhibitory effect observed upon application of high ABA concentrations is an ethylene-dependent process (Ghassemian et al., 2000), while the promoting effect of low ABA concentrations is ethylene independent (Li et al., 2017). Auxin was shown to regulate root development in response to both high and low ABA concentrations. Roots of auxin signaling and influx mutants (*iaa7/axr2* and *aux1*, respectively) were less responsive to exogenous ABA regardless its concentration, indicating that both ABA effects are regulated by auxin. Additionally, Pin-formed 2 (PIN2), an important auxin efflux transporter expressed in the lateral root cap, is also involved in ABA response (Li et al., 2017).

Abscisic acid regulates root development also through the action of microRNAs (miRNAs), short single-stranded, small noncoding RNAs that play a major role in plant growth and development, as well as in hormone and stress response (Yan et al., 2017). In *Populus euphratica*, ABA modulates root growth through miRNA-mediated pathway, together with other hormones, such as GA and brassinosteroids (BRs) (Lian et al., 2018). For instance, peu-miRNA477 was shown to target the repressor of GA1-like (RGL1), a negative regulator of gibberellin response. The *rgl1* mutant line showed a dwarf shoot phenotype with an increased lateral root biomass, revealing the importance of RGL1 in plant development. Interestingly, treatment of wild-type plants with exogenous ABA resulted in an increase in peu-miRNA477 expression level, in both shoots and roots, accompanied by a remarkable phenotype, closely related to that of *rgl1* mutant: repressed shoot and promoted root growth. The second example links ABA with BR as BR-insensitive 1 (*BRI1*) gene was shown to mediate BR signaling response in root growth through interaction with BAK1 (BR-insensitive 1–associated receptor kinase 1 related). Exogenous ABA treatment led to a decrease in the level of peu-miRNA-n68 that targets BAK1, resulting in an induction in BAK1 expression and enhanced root growth (Lian et al., 2018).

### ABA Transport and Stomata Closure

In higher plants, water is absorbed from soil by the root system and is transported to the shoot where it transpires. Abscisic acid regulates the water status in plants by regulating the closure of stomata that mediate uptake and release of oxygen and carbon dioxide for respiration and photosynthesis (Jia and Zhang, 2008; Manzi et al., 2016). Moreover, stomata play an important role in pathogen defense, as they are considered as a port of entry of pathogens including bacteria, fungi, and viruses. By regulating stomata aperture and inducing the expression of defense-related genes, ABA initiates plant’s defenses against pathogen attack (Lim et al., 2015). Under mild water limitation, when the soil starts to dry, ABA accumulates in root tissues leading to the decrease of stomatal conductance, while under drought stress, ABA accumulates in xylem. This distribution indicates that ABA is synthesized in roots, released to xylem vessels, and transported to the shoots. However, ABA is not only synthesized in roots, as ABA biosynthesis genes, e.g. NCED2, ABA2, AAO3, are also expressed at the vascular bundle in leaves where the guard cells are located at the epidermal layer, suggesting that ABA is transported between leaf tissues to elicit stomata response (Kuromori et al., 2018). In *Arabidopsis*, the two ATP-binding cassette (ABC) transporter proteins ABCG25 and ABCG40, which are localized at the plasma membrane in vascular tissues and guard cells, respectively, have been shown to modulate stomata closure and ABA sensitivity (**Figure 2**) (Kang et al., 2010; Kuromori et al., 2010). Overexpression of AtABCG25 led to enhanced stomata response, supporting the hypothesis that ABA is synthesized in the vascular tissues and transported to the guard cells (Kuromori et al., 2016). The *Arabidopsis* nitrate transporter 1/peptide transporter family (NPF4.6) is a further ABA transporter that is expressed in vascular tissues, suggesting a complementary role with AtABCG25 and AtABCG40 in modulating the amount of transported ABA from vascular cells to guard cells (Kuromori et al., 2018). Finally, ATDTX50, a member of multidrug and toxin efflux transporter family, was identified as an ABA exporter localized at the plasma membrane and expressed in vascular tissues and guard cells (Zhang et al., 2014). The corresponding mutant line *dtx50* showed retarded growth, which might be linked to high ABA sensitivity or high endogenous ABA level (Kuromori et al., 2018).

## Strigolactones

Strigolactones are carotenoid-derived plant phytohormones that regulate different aspects of plant development, including shoot branching, establishing of root architecture, and leaf senescence, and are involved in biotic and abiotic stress response (Xie et al., 2010; Al-Babili and Bouwmeester, 2015; Decker et al., 2017; Saeed et al., 2017; Jia et al., 2019b). However, SLs were originally identified as inducer of root parasitic plant seed germination of genera *Striga*, *Orobanche*, *Alectra*, and *Phelipanche* (Xie et al., 2010). These weeds are a major threat for global food security, causing enormous yield losses in yields of cereals and many other crops (Parker, 2009; Parker, 2012;Atera et al., 2011;Bouwmeester et al., 2003; Pennisi, 2015). Later on, it was shown that SLs act as a chemical signal released into the soil to attract arbuscular mycorrhizal (AM) fungi for establishing the beneficial AM symbiosis (Akiyama et al., 2005;Lopez-Raez et al., 2015). Approximately 80% of land plants form this symbiosis that provides the AM fungi (AMF) with photosynthetic products and the plant with water and important micronutrients with low mobility in soil, especially phosphorus (Bonfante and Genre, 2010; Gutjahr and Parniske, 2013; Lanfranco et al., 2017; Yoneyama et al., 2019); therefore, SL biosynthesis and release are induced upon phosphate deficiency (Yoneyama et al., 2012).

### SL Biosynthesis

There are around 25 characterized natural SLs so far, which are classified based on their chemical structure in canonical and noncanonical SLs. Canonical SLs consist of a butenolide lactone ring (D ring) linked by enol ether bridge in *R*-configuration (2′*R*), to a tricyclic lactone (ABC ring). Canonical SLs are divided into strigol- and orobanchol-like SLs based on the stereochemistry of B-/C-ring junction, whereas in the strigol-type SLs the C-ring is in β-orientation (up; 8b*S*-configuration), while in orobanchol-type SLs, the C-ring is in α orientation (down; 8b*R*-configuration) (Jia et al., 2017). Noncanonical SLs contain the butenolide lactone ring (D ring) and the enol ether bridge in *R*-configuration, which are characteristic for all-natural SLs. However, they have, as a second moiety, different structures instead of the ABC ring. Examples for noncanonical SLs are methyl carlactonoate, heliolactone, zealactone and zeapyranolactone, and avenaol (Al-Babili and Bouwmeester, 2015; Jia et al., 2017; Jia et al., 2019b; Koltai and Prandi, 2019). The diversity of SLs is a result of the structural variability of noncanonical SLs and modifications of the A and B rings in canonical ones, which include methylation, hydroxylation, ketolation, and epoxidation (Jia et al., 2017; Wang and Bouwmeester, 2018; Jia et al., 2019b).

**Figure 3:**
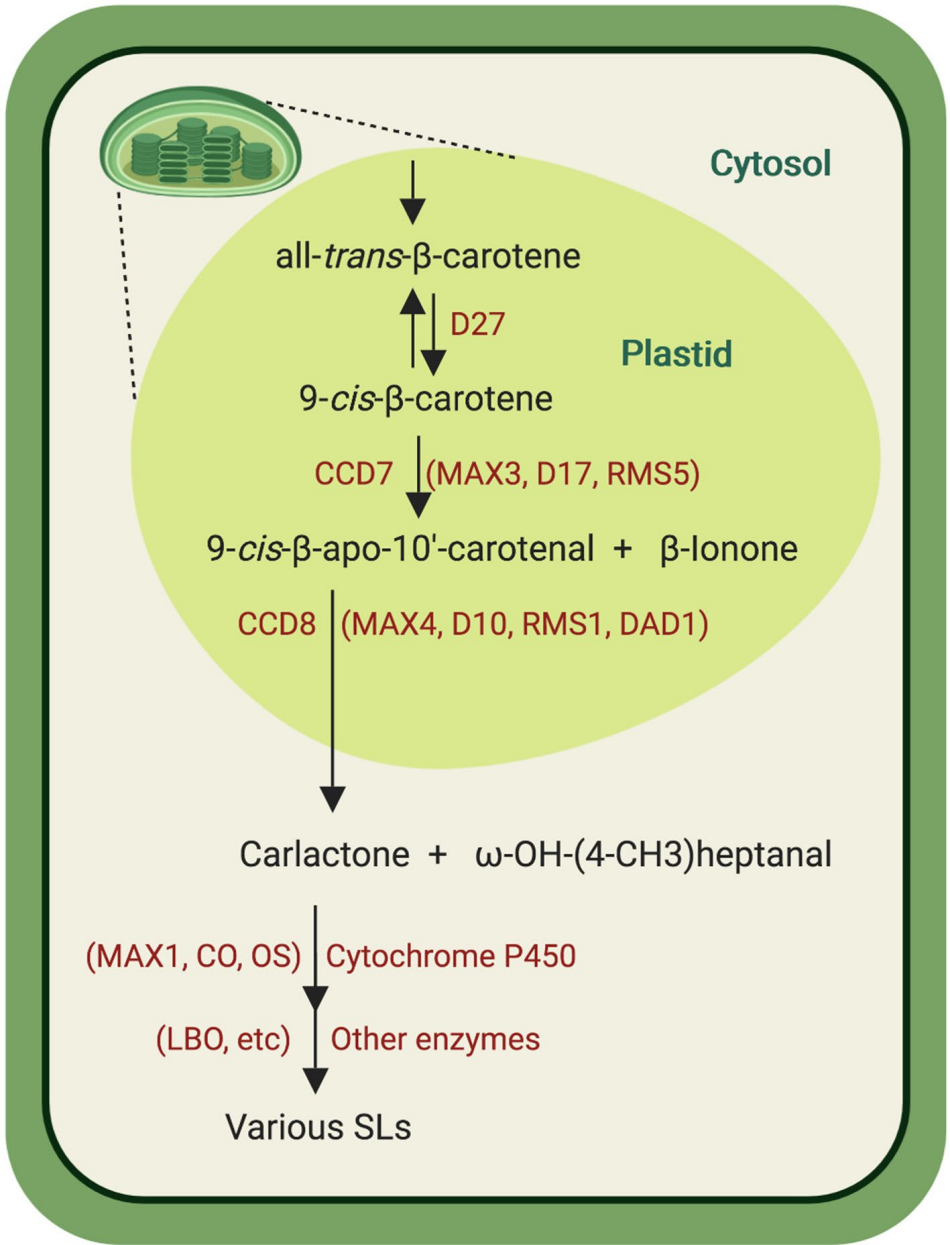
Strigolactones biosynthesis pathway. The conversion of all-*trans*-β-carotene into carlactone is successively catalyzed by the *cis*/*trans*-β-carotene isomerase D27 that reversibly converts all-*trans*-β-carotene into 9-*cis*-β-carotene, which is subsequently cleaved by CCD7 into 9-*cis*-β-apo-10′-carotenal and β-ionone. The next enzyme, CCD8, transforms 9-*cis*-β-apo-10′-carotenal into carlactone and ω-OH-(4-CH3) heptanal. Carlactone is then oxidized, catalyzed by CYP711 enzymes [*Arabidopsis* MAX1, rice carlactone oxidase (CO), and orobanchol synthase (OS)] into carlactonoic acid and other SLs. After methylation, carlactonoic acid is oxidized by the oxidoreductase LBO into yet unidentified product. See text for abbreviations. Created with ‘Biorender’.

The similarity between the SL A-ring and the ionone ring in carotenoids led to the hypothesis that SLs might, like ABA, originate from carotenoids. This hypothesis was confirmed by investigating the SL release in different carotenoid-deficient mutants and through application of inhibitors of carotenoid biosynthesis (Matusova et al., 2005). However, SL biosynthesis remained elusive for many years because of lack of knowledge about the involved enzymes. The discovery that more branching/high tillering mutants from different plant species are SL deficient led to the breakthrough discovery of the SL hormonal function and provided candidate for biosynthesis enzymes and perception components, enabling the elucidation of major steps of SL biosynthesis and signal transduction. Strigolactone biosynthesis is initiated by the reversible conversion of the all-*trans*-β-carotene (C_40_) into 9-*cis*-β-carotene in the plastids, which is catalyzed by the 9-*cis*/*trans*-all-β-carotene isomerase Dwarf27 (D27) (Alder et al., 2012; Waters et al., 2012; Bruno and Al-Babili, 2016; Abuauf et al., 2018) (**Figure 3**). The resulting 9-*cis*-β-carotene is cleaved by the carotenoid cleavage dioxygenase 7 (CCD7) (*Arabidopsis* MAX3, rice D17/HTD1, pea RMS5, and petunia DAD3), a stereospecific enzyme that converts only 9-*cis*-configured carotenoids into 9-*cis*-β-apo-10′-carotenal (C_27_) and β-ionone (C_13_) (Bruno et al., 2014). 9-*cis*-β-apo-10′-carotenal is the substrate of CCD8 (*Arabidopsis* MAX4, rice D10, pea RMS1, and petunia DAD1), an unusual CCD that catalyzes a combination of different reactions leading to the central SL biosynthesis intermediate carlactone (CL) and ω-OH-(4-CH3) heptanal (Alder et al., 2012; Bruno et al., 2017; Guillotin et al., 2017). Following its formation in plastids, CL is then exported into the cytosol where it is further converted by cytochrome P450 enzymes (CYP) of the 711 clade, e.g. the *Arabidopsis* MAX1 and the rice carlactone oxidase (Zhang et al., 2014). Recent investigation of the activity of CYP711 enzymes from different plant species allowed the classification of these enzymes into three types, types A1, A2, and A3 (Yoneyama et al., 2018). In *Arabidopsis*, CL is hydroxylated and oxidized at C19 into carlactonoic acid (CLA) by AtMAX1 (a CYP711A1 type). Carlactonoic acid is substrate of an unidentified methyltransferase that forms methyl carlactonoate (MeCLA) (Abe et al., 2014; Jia et al., 2017; Yoneyama et al., 2018). Recently, the enzyme lateral branching oxidoreductase (LBO), a member of the 2-oxoglutarate and Fe^II^-dependent dioxygenase family, was identified as a further SL biosynthesis enzyme downstream of MAX1. *In vitro* studies indicated that LBO converts MeCLA into an unidentified product with an additional oxygen atom (Brewer et al., 2016). Rice genome encodes five MAX1 homologs (Os900, Os1400, Os1500, Os1900, Os5100). *In vitro* studies and transient expression in *Nicotiana* *benthamiana* suggest that Os900 (a CYP711A2 type) catalyzes the repeated oxygenation and ring closure in CL to yield 4-deoxyorobanchol (4DO), a canonical SL. Os1400, a rice CYP711A3 type enzyme, catalyzes the hydroxylation of 4DO into orobanchol (Sun et al., 2014). It was also shown that the three rice CYP711, Os1400, Os5100, and Os1900, can also convert CL into CLA, indicating that CLA is a common component in plant SL biosynthesis (Yoneyama et al., 2018). A recent study revealed that CCD7 and CCD8 can also utilize hydroxylated substrates, forming 3-OH-CL as a further product of the SL biosynthesis core pathway. 3-OH-CL might be the precursor of yet unidentified SLs, which contributes to SL diversity (Baz et al., 2018).

### SL Transport

Grafting is frequently used to investigate hormone and metabolite fluxes between shoots and roots. Using this technique, it was shown that the more branching phenotype of SL biosynthesis mutants scions can be rescued by wild-type root stock, suggesting the presence of a root to shoot SL transport (Leyser, 2009; López-Ráez et al., 2010). Strigolactones are also released into the rhizosphere, which suggests the presence of a further transport system. In *Petunia*, this release was shown to be mediated by an ABC transporter protein pleiotropic drug resistance 1 (PDR1) (Kretzschmar et al., 2012), acting as a SL transporter. Pleiotropic drug resistance 1 is localized in the plasma membrane of the cortex cells in root tip and of the hypodermal passage cells, the passage points of the AMF. The latter localization indicates that PDR1 may guide the hyphae of AMF to the hypodermal passage cells (Jia et al., 2019b).

### SL Signal Transduction

Strigolactone perception is initiated by binding to the SL receptor, Dwarf14 (D14), a α/β-hydrolase superfamily protein, which contains a conserved Ser-His-Asp catalytic triad required for SL hydrolysis and signal transduction (Hamiaux et al., 2012; Nakamura et al., 2013; Waters et al., 2015). In the absence of SLs, this receptor exhibits an open solvent-exposed ligand-binding pocket. Binding of the SL ligand in the pocket triggers its hydrolysis into the ABC ring and an intermediate D-ring moiety, which is followed by releasing the ABC ring and covalent binding of the D-ring to form the so-called covalently linked intermediate molecule (CLIM), accompanied by a conformational change of D14. These processes are critical for SL signal transduction that proceeds through binding to the F-box protein MAX2 (D3 in rice) and recruiting target expression repressors, such as the rice D53 or members of the *Arabidopsis* SMXL proteins for ubiquitination and proteasome-mediated degradation. (Saeed et al., 2017; Waters et al., 2017; Jia et al., 2019b). Most recently, structure-function studies of the rice D14-D3-D53 complex show that D3 possesses a C-terminal α-helix (CTH), which can occur in two conformation states, i.e. an engaged form and a dislodged form. The engaged form facilitates the binding of D3 and D14 in its CLIM form, whereas the dislodged form recognizes unmodified D14 and inhibits its enzymatic activity. The D3 CTH enables D14 to recruit D53 in SL presence, which then triggers SL hydrolysis. This structural plasticity of the SCF^D3^–D14 ubiquitin ligase suggests a model that explains how D3 coordinates SL perception and hydrolysis (Shabek et al., 2018).

### SL and Shoot Architecture

Strigolactone is one of the key hormones in determining shoot architecture. In fact, the discovery of SLs as a new plant hormone was achieved through characterizing a series of more branching or high tillering mutants in different species. These mutants include more axillary growth (*max*) in *Arabidopsis* (Sorefan et al., 2003; Booker et al., 2004; Booker et al., 2005; Stirnberg et al., 2007), *ramosus* (*rms*) in pea (Beveridge et al., 2000; Morris et al., 2001; Sorefan et al., 2003; Foo et al., 2013), *dwarf* (*d*)/high-tillering dwarf (*htd*) in rice (Ishikawa et al., 2005; Arite et al., 2007; Arite et al., 2009; Lin et al., 2009; Zhou et al., 2013), and decreased apical dominance (*dad*) in petunia (Snowden et al., 2005; Simons et al., 2007; Drummond et al., 2009; Drummond et al., 2012; Hamiaux et al., 2012). This branching phenotype was resolved at the molecular level through SL perception mechanism in vascular plants. As previously mentioned, SL perception requires α-β hydrolase D14 and MAX2 and leads to (i) the cleavage of SL and (ii) the proteasome-mediated degradation of D53 and SMXL6/7/8 in rice and *Arabidopsis*, respectively, in order to regulate shoot branching. Indeed, it was shown that the *smxl6 smxl7 smxl8* triple mutants could restore the branching and SL level to wild type (Soundappan et al., 2015).

**Figure 4:**
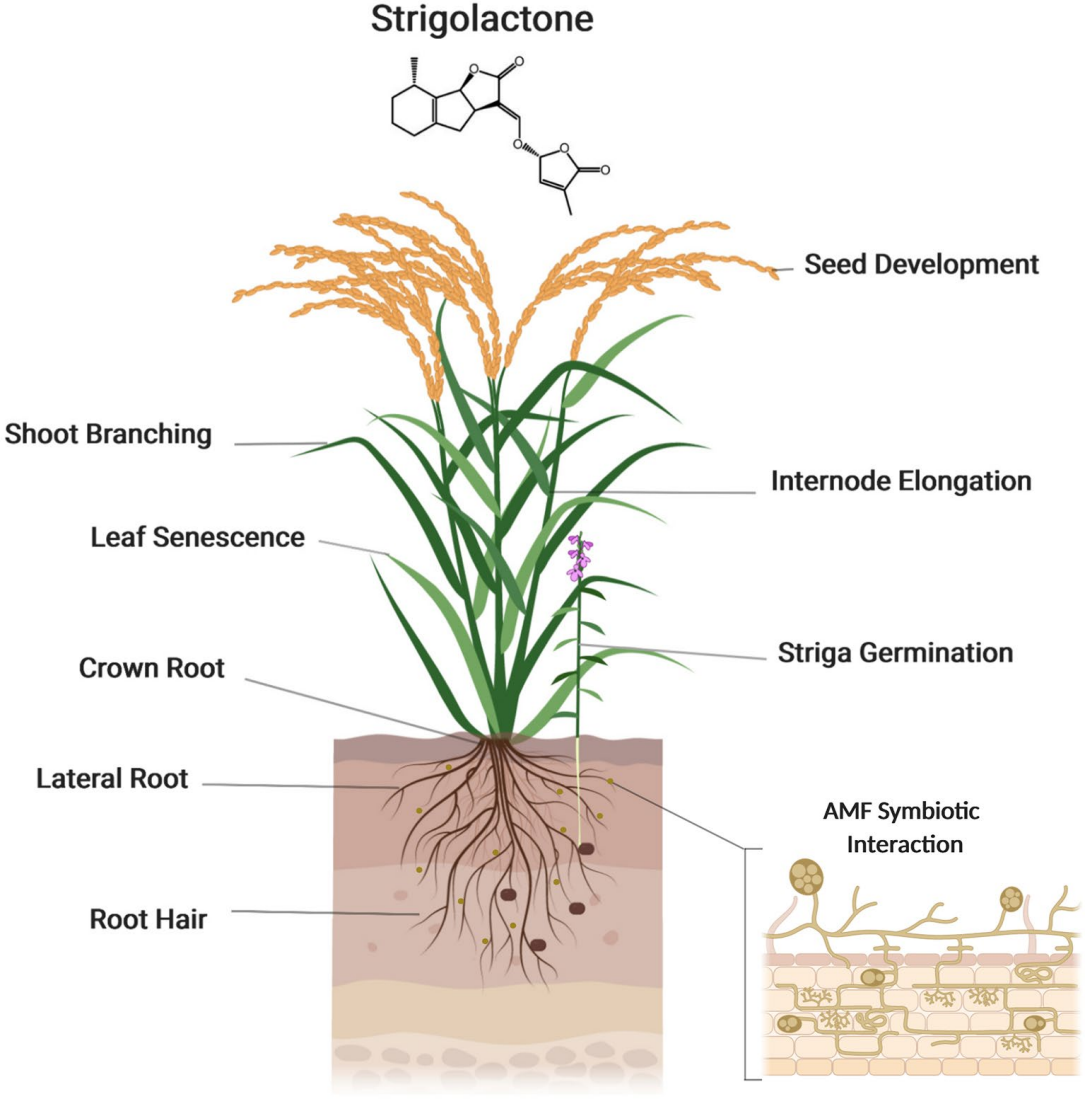
Strigolactones (SLs) function in plant growth and development. Strigolactones regulate several developmental processes, such as shoot branching, seed development, internode elongation, and leaf senescence. Strigolactones are also a key regulator of root architecture, modulating the growth of crown and lateral roots and root hairs. Created with ‘Biorender’.

Auxin is a major regulator of shoot branching. Many lines of evidence demonstrate that SLs interact with auxin to regulate this developmental process (Leyser, 2009). It has been shown that SLs regulate auxin homeostasis by modulating polar auxin transport (PAT) that is supposed to determine axillary bud dormancy/release in *Arabidopsis*. On the other hand, auxin controls the expression of SL biosynthesis genes and hence determines SL biosynthesis. Moreover, the negative feedback exerted by SLs on their own biosynthesis is mediated by auxin. In *Arabidopsis*, it was proposed that the inhibitory effect of SLs on shoot branching is caused, at least partially, by a reduction of PAT from the axillary buds to shoots by down-regulating the expression of the auxin transporter, PIN1. In addition, local auxin transport between the PAT stream and surrounding tissues, which is called connective auxin transport, rather than PAT itself, was shown later to be essential for the initial growth of axillary buds initial growth, while PAT is essential for their sustainable growth in both in *Arabidopsis* and pea (Leyser, 2009; Barbier et al., 2019). Sucrose also plays an important role in regulating bud outgrowth, by interacting with plant hormones: sucrose induces the expression of auxin biosynthesis genes and promotes the accumulation of cytokinin accumulation, which triggers buds outgrowth (Barbier et al., 2019) (**Figure 4**).

### SLs in Root Development

In addition to shoot architecture, SLs play an important role in root development. The rice SL biosynthesis (*d10, d17* and *d27*) and perception (*d3* and *d14*) mutants exhibit shorter crown roots and contain a lower number of root meristem cells, compared to wild-type plant (Arite et al., 2012). In barley, the SL receptor mutant *d14* mutant shows an increase in lateral root density and shorter seminal roots (Marzec et al., 2016). In *Arabidopsis*, treatment with the SL analog *rac*-GR24 leads to a reduction in the number and growth of lateral roots in a MAX2-dependent manner, while *max2* mutant, but not SL biosynthesis mutants, shows a strong increase in lateral root density (Kapulnik et al., 2011a; Ruyter-Spira et al., 2011) due to SL insensitivity. SMXL6,7,8 might act downstream of MAX2, as the *smxl6,7,8* triple mutants shows less lateral root density compared to wild type, while the *smxl6,7,8* in *max2* mutant background showed ∼50% decrease in lateral root density, suggesting that SMXLs are growth regulators thus repressed by SL signaling (Soundappan et al., 2015).

Strigolactones also regulate root hair elongation, as shown by treatment of tomato and *Arabidopsis* plants with rac-GR24. Confirming the specificity of this effect, application of rac-GR24 to *Arabidopsis* *max2* mutant did not promote root hair elongation (Kapulnik et al., 2011a; Kapulnik et al., 2011b; Matthys et al., 2016). Strigolactones also inhibit adventitious root formation (Rasmussen et al., 2012). The effect of SLs on root development largely depends on auxin (Ruyter-Spira et al., 2011).

### SLs in Leaf Senescence

Leaf senescence is a final phase of plant development. This process starts with the degradation of proteins and lipids into small-molecular-weight compounds and relocation of nutrients to younger cells or seeds (Guo and Gan, 2005). Leaf senescence is induced by several factors such as stress, aging, darkness, and plant hormones including SLs. Thus, *Arabidopsis* more axilliary branches 2/ORESARA 9 (MAX2/OREA9) gene was initially identified as a leaf senescence gene (Stirnberg et al., 2007). Mutation in the rice homolog D3 leads also to delayed leaf senescence (Yan et al., 2007), and a similar phenotype was observed in the corresponding petunia and *Lotus japonicas* mutant (Liu et al., 2013). Providing a further evidence for the positive role of SLs in leaf senescence, exogenous application of *rac*-GR24 accelerates leaf senescence in rice wild-type and SL biosynthesis mutants, i.e. *d27*, *d17*, and *d10*, but not in seedlings of the SL signaling mutants *d14* and *d13* (Ali et al., 2018). Similarly, the *Arabidopsis* SL-deficient and SL-insensitive mutants show delayed leaf senescence (Ueda and Kusaba, 2015).

## Novel Carotenoid-Derived Signaling Molecules

In the last years, there has been large progress in identifying novel carotenoid-derived signaling molecules and regulatory metabolites. In the following, we touch on the exciting apocarotenoid volatile cyclocitral and its functions in regulating stress response and determining root architecture. We also briefly describe the recently discovered regulatory metabolites, anchorene, and zaxinone and their role in plant development.

### β-Cyclocitral

**Figure 5:**
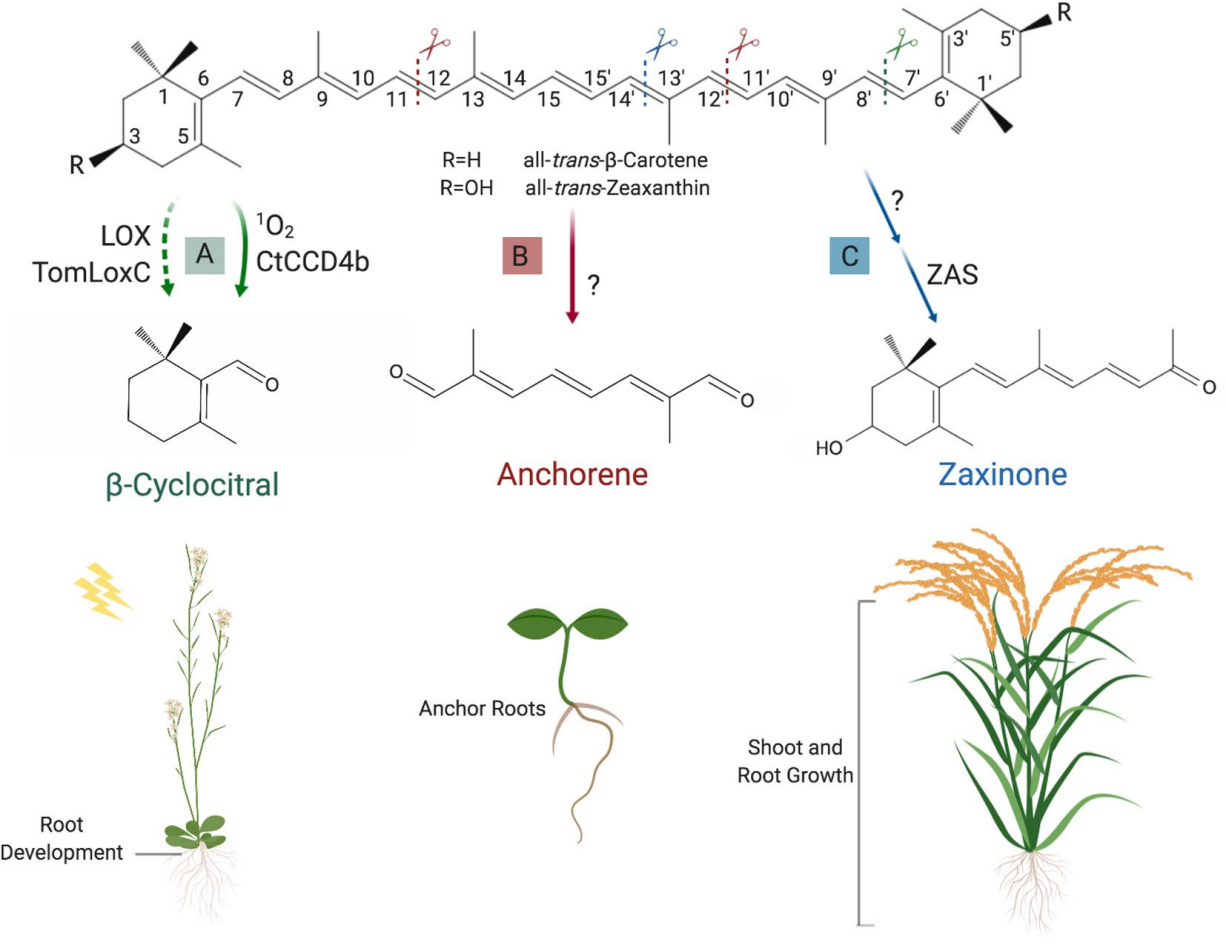
Formation and function of novel regulatory apocarotenoids. **(A)** β-Cyclocitral can be produced through cleavage of all-*trans*-β-carotene at the (C7′–C8′) double bound, which is catalyzed by CtCCD4b or ^1^O_2_ attack. Very recently, it was also shown that lipoxygenases, e.g. the tomato TomLoxC and the *Arabidopsis* LOX, can mediate the formation of this volatile, which acts as a growth regulator of roots and is involved in high light acclimation. **(B)** Anchorene, which can be formed by cleaving the (C11–C12) and (C11′–C12′) double bounds in all carotenoids downstream ζ-carotene in the carotenoid pathway, stimulates ANRs formation in *Arabidopsis* seedlings. **(C)** The recently discovered zaxinone is synthesized from all-*trans*-zeaxanthin (or all-*trans*-lutein) *via* the intermediate apo-10′-zeaxanthinal (not shown). The latter is the substrate of Zaxinone synthase (ZAS) that mediates the cleavage of the (C13–C14) double bond. Zaxinone is required for normal rice growth and development and is a negative regulator of SL biosynthesis. Created with ‘Biorender’.

β-Cyclocitral is a β-carotene cleavage product that can be formed by enzymes from a subgroup of the CCD4 clade (Rodrigo et al., 2013) (**Figure 5A**) and, non-enzymatically, through attack by singlet oxygen (^1^O_2_) (Ramel et al., 2012a). Very recently, Gao et al. (2019) showed that the amount of β-cyclocitral (and other carotenoid-derived volatiles) was proportional to the LOX 13S-lipoxygenase expression level; a decrease of apocarotenoid content was observed in TomLocX antisense transgenic tomato and in AtLOX *Arabidopsis* mutant, compared to wild type. This study confirms previous *in vitro* observations where carotenoids were shown to act as a cosubstrate for these enzymes during the fatty acid oxidation (Hayward et al., 2017) and reveals the existence of an alternative route for apocarotenoid biosynthesis in planta. β-Cyclocitral has been detected in many plant species, including tomato (Tieman et al., 2012), rice (Hinge et al., 2016), parsley (Linde et al., 2016), tea (Ojha and Roy, 2018), grape (Chen et al., 2017), various trees (Gómez and Ledbetter, 1997; Tewari et al., 2015), and moss (Saritas et al., 2001). Studies in *Arabidopsis* showed that β-cyclocitral is a likely second messenger that conveys the ^1^O_2_ stress signal to the nucleus, altering the transcription of ^1^O_2_-regulated genes *via* methylene blue sensitivity protein, a small zinc finger protein (Ramel et al., 2012b; Shumbe et al., 2017). Similar activity was also reported for dihydroactinidiolide, a volatile resulting from the oxidation of β-ionone by ^1^O_2_ (Ramel et al., 2012b) (for a recent review, see D’Alessandro and Havaux, 2019). The transcriptome reprograming process, triggered by β-cyclocitral, increases the plant tolerance to photo-oxidative stress and initiates acclimation to high light conditions (Ramel et al., 2012b). Recently, it was shown that high light leads to a significant increase in the content of glycosylated β-cyclocitral (Mi et al., 2019b), which might be a mechanism for deactivation of this signaling molecule (D’Alessandro and Havaux, 2019).

A very recent study underpins the biological importance of β-cyclocitral by revealing its role as root growth regulator (Dickinson et al., 2019). Assuming that unknown apocarotenoid signals are required for root branching and lateral roots emergence, the authors used a targeted chemical genetic approach to identify apocarotenoids that enhance root branching. To increase the sensitivity of the roots by minimizing the level of endogenous apocarotenoids, they performed this screen in the presence of D15, an inhibitor of CCDs, at a concentration that reduces lateral roots emergence by 50% (Dickinson et al., 2019). Treatment with β-cyclocitral and dihydroactinidiolide led to significant increases in lateral root branching, with β-cyclocitral showing the strongest effect. In addition, application of β-cyclocitral in the absence of D15 also stimulated the growth of primary roots. Investigation of β-cyclocitral effect at cellular level showed that this compound promotes cell division in undifferentiated cells in meristems of lateral and primary roots. This effect is independent of ROS, auxin, and BR signaling pathways, as shown by using corresponding hormone-responsive marker lines and mutants.

Application of β-cyclocitral to tomato and rice seedlings demonstrated that this apocarotenoid is a conserved root growth regulator across plant species. The positive effect of β-cyclocitral was also observed under salt stress conditions: applied to salt-stressed rice roots rescued the negative effect of salt on root depth and showed in salty soil a positive effect on the vigor of rice plants (Dickinson et al., 2019).

### Anchorene

Carotenoids can be targeted by repeated oxidative cleavage, which yields dialdehyde products (diapocarotenoids), besides monocarbonyl apocarotenoids. However, carotenoid-derived dialdehydes have attracted little attention because of their instability that makes their detection in biological systems and the identification of their possible biological activity difficult. Indeed, diapocarotenoids were mainly investigated because of their role as precursor of crocin and bixin, two pigments accumulated in saffron stigma and bixa seeds. (Giuliano et al., 2003; Frusciante et al., 2014; Demurtas et al., 2018). Recently, Jia et al. established a screening system to identify known and predicted diapocarotenoids involved in plant development, by assaying alterations in *Arabidopsis* roots. This approach led to the discovery of the presumed diapocarotenoid anchorene as a novel carotenoid-derived bioactive that promotes the development of anchor roots (ANRs) (Jia et al., 2019a), a less investigated type of *Arabidopsis* roots, which develop from the collet region situated at the root hypocotyl junction (Lucas et al., 2011). Although it is still unclear how it is formed, anchorene (C_10_) is a natural metabolite, as confirmed by LC-MS analysis (Jia et al., 2019a; Mi et al., 2019a), and its structure indicates that it can be produced by cleaving the (C11–C12) and (C11′–C12′) double bound from all carotenoids downstream of ζ-carotene in the carotenoid biosynthesis pathway (**Figure 5B**). In *Arabidopsis*, application of anchorene induces ANR formation. The regulation of ANR by anchorene is highly specific to its chemical structure, as application of a structural isomer did not show an effect on ANR development, and altering anchorene functional group(s) to alcohol, acid, or acid-ethyl ester resulted in a loss of activity (Jia et al., 2019a). The usage of auxin reporter lines and an auxin transport inhibitor, together with transcriptome analysis, showed that anchorene’s effect on ANR development is caused by modulating auxin homeostasis. Indicating their role in increasing nitrogen uptake, Jia et al. showed that ANR formation is triggered by nitrogen deficiency. Interestingly, lack of nitrogen led also to an increase in anchorene content, pointing to a possible function in regulating plant’s response to this adverse growth condition (Jia et al., 2019a).

### Zaxinone

A recent study unraveled a sixth new clade in the plant CCD family, which is present in many plant species but not in *Arabidopsis* or other members of *Brassicacea*. *In vitro* characterization of a rice representative of this clade showed that it cleaves apo-10′-zeaxanthinal (3-OH-β-apo-10′-carotenal, C_27_) at the (C13–C14) double bound, resulting in a C_18_-ketone and an instable dialdehyde (C_9_) (Wang et al., 2019). Wang et al. called the C_18_ apocarotenoid zaxinone and, accordingly, the corresponding enzyme ZAS (**Figure 5C**). They also confirmed that zaxinone is a natural metabolite present in several plant species, including rice (Wang et al., 2019; see also Mi et al., 2018). Analysis of a corresponding *zas* mutant in comparison to wild type showed that it has similar zaxinone content in shoots but contained less amounts of this compound in roots, the tissue with the highest ZAS expression levels. These results confirm the enzymatic activity of ZAS, but also demonstrate that zaxinone can be formed *via* a route(s) that does not involve ZAS. The lower zaxinone content in *zas* roots mutant was accompanied by a reduced crown root length and number, a decrease in root and shoot biomass, and a lower tiller and panicle number. Taking into consideration that low tillering phenotype might be a result of increased SL biosynthesis, Wang et al. determined SL content in root tissues and exudates and found that *zas* mutant produced and released higher amounts of SLs. Accordingly, *zas* root exudates were more active in inducing *Striga* germination, compared to wild type. Exogenous application of zaxinone rescued the root phenotypes in *zas* mutant and increased root growth in wild-type seedlings, suggesting that this metabolite is growth promoting and required for normal rice growth and development. Moreover, zaxinone’s application led to a decrease in SL content and release, demonstrating that zaxinone is a negative regulator of SL biosynthesis. Expression analysis of SL biosynthetic genes showed that zaxinone acts at transcript level. Interestingly, application of zaxinone to a *Striga* susceptible rice variety alleviated the infestation by this root parasitic plant, indicating an application potential of zaxinone in combating *Striga*, besides the possibility of employing it to promote rice growth (Wang et al., 2019).

## Conclusion

Plant growth and development, as well as response to environmental changes and stress factors, are regulated by a set of hormones and signaling molecules, which includes metabolites originating from carotenoids. The initial step in the formation of these carotenoid-derived signals is an oxidation step that yields shortened, carbonyl-group containing molecules called apocarotenoids. Some apocarotenoids can immediately act as signaling molecules, e.g. β-cyclocitral, or growth regulators, e.g. zaxinone and anchorene. Further modifications of the primary cleavage products lead to the known hormones ABA and SLs. It can be expected that future work will unravel new carotenoid-derived regulatory metabolites and that carotenoid metabolism will remain an intriguing and important research field in plant science, connecting photosynthesis, the primary metabolic process, with plant growth and development.

## Author Contributions

This review was written by AF and JB. SA-B supervised the writing of the review and was involved in its further editing and revision. MZ contributed to the editing and organization of the review.

## Funding

This work was supported by baseline funding and Competitive Research Grant (CRG2017) given by King Abdullah University of Science and Technology (KAUST) to SA-B, and by the Deutsche Forschungsgemeinschaft (DFG, German research Foundation) under Germany’s Excellence Strategy EXC 2048/1-390686111.

## Conflict of Interest

The authors declare that the research was conducted in the absence of any commercial or financial relationships that could be construed as a potential conflict of interest.
